# Minimally Invasive Carpal Tunnel Release: A Technical Note and a 20-Year Retrospective Series

**DOI:** 10.7759/cureus.21426

**Published:** 2022-01-19

**Authors:** Ignazio Gaspare Vetrano, Grazia Devigili, Vittoria Nazzi

**Affiliations:** 1 Department of Neurosurgery, Fondazione IRCCS Istituto Neurologico Carlo Besta, Milan, ITA; 2 Neurological Unit, Fondazione IRCCS Istituto Neurologico Carlo Besta, Milan, ITA

**Keywords:** nerve surgery, minimally invasive, median nerve, entrapment, carpalotome, carpal tunnel release

## Abstract

Introduction

The surgical treatment of carpal tunnel syndrome (CTS) has been enriched, during the last years, by different minimally invasive techniques to decompress the median nerve at the wrist as the endoscopic approaches or modified open technique. However, controversy remains about their safety and complication rate. We present the results of our minimally-invasive technique to median nerve release at the wrist. We will discuss the instrumental preoperative assessment, surgical steps, post-operative management, and complications.

Methods

We retrospectively reviewed clinical and neurophysiological data of all patients admitted at our institution between January 2001 and December 2020 for CTS surgery. The technique, performed under local anesthesia, is based on a single, small, linear transverse incision proximal to the wrist fold. After unsharpened dissection of subcutaneous tissues, a grooved guide is inserted in a slightly medial direction towards the fourth finger; this strategy prevents possible damages of nerve branches that could originate at this level. A second small incision over the guide’s tip allows a wide corridor in the context of the ligament. The carpalotome is then inserted into the guide; the two minor wounds are closed with 5-0 prolene sutures. The final result is a wide release of the nerve.

Results

A total of 1568 operations on 1371 patients were performed using the described technique at our institution. The patients’ cohort showed a higher prevalence of women (68%), with a mean age of 56.4 years (range 24-88 years). Paresthesia and numbness of the first three fingers were the most frequent signs and symptoms. All patients were submitted to a preoperative electrophysiological evaluation, which revealed the typical signs of CTS in most patients. The US evaluation of the median nerve at the wrist was a more recent introduction, dating from 2018. In 47 patients, despite an electromyography (EMG) not showing marked neurophysiological signs of severe CTS, the ultrasonographic evaluation was strongly consistent with the clinical diagnosis. In such patients, carpal tunnel release determined the resolution of symptoms. In 99.8% of total cases, we obtained a complete symptoms remission, with the disappearance of acroparesthesia and numbness.

Conclusion

The use of this technique has become widespread at our institution due to fewer local complications, a very low rate of recurrence, faster functional recovery, and reduced surgical time if compared to traditional open surgery and to endoscopic release too.

## Introduction

The entrapment of the median nerve at the wrist, which determines carpal tunnel syndrome (CTS), is the most frequent neuropathy, with an estimated prevalence of up to 4-7% in the general adult population [1]. Therefore, carpal tunnel release (CTR) is among the most frequent hand surgery procedures performed worldwide [2]. The traditional open technique requires a longitudinal incision extending between the distal end of the transverse carpal ligament (TCL) and the proximal end of the palmar crest [3]. Whereas this access provides a complete exposure of the median nerve, it could carry the risk of scar tissue and scar sensitivity, along with a possible flexion contracture in the wrist. All these conditions can significantly delay a complete functional recovery [4,5]. To avoid such complications, less invasive techniques have been proposed, such as the endoscopic-assisted release or mini-palmar incision surgery, sometimes by using innovative surgical instruments [6,7]. When adopting a less invasive procedure, the final aim is to reduce surgical time and possible complications (including the extent of the incision and post-operative scar), providing faster recovery and an earlier return to work and daily activities. Despite these premises, all the techniques mentioned above could determine several complications, such as vascular, nerve, and tendon damages, or the incomplete release of the transverse ligament, leading to recurrence [8].

Moreover, although the diagnosis of CTS is mainly clinical, coupling neurophysiological evaluation with ultrasonographic (US) imaging can increase the diagnostic accuracy and indications for surgery with high concordance to clinical scores and nerve conduction studies [9]. Moreover, in the case of diagnostic uncertainty, symptoms not localized, severe and diffuse pain, US can help discriminate CTS secondary to ganglia or space-occupying lesions. However, the US preoperative evaluation for median nerve entrapment has not a widespread diffusion [10]. CTR could appear as a simple surgical procedure, but it could determine morbidity due to nerve or vascular damage, mainly in the case of anatomical variants. A preoperative morphological US evaluation can reduce the risk of damages.

We present a technical note about the preoperative management and the surgical technique for CTR, based on Franzini carpalotome (a modified Paine retinaculotome), with only two short cutaneous incisions at the wrist and palm. We will discuss the instrumental preoperative assessment, surgical steps, post-operative management, and complications, and the results of this technique in a patient cohort submitted to surgery during the last 20 years.

## Materials and methods

We retrospectively reviewed clinical and neurophysiological data regarding all patients admitted at our institution during the last two decades. A dedicated database is employed to collect such reports prospectively. We included in the study all consecutive patients who were surgically treated for CTS with our minimally invasive technique between January 2001 and December 2020 at our institution, which represents a tertiary, national referral center with high-volume os surgical procedures on the peripheral nervous system. Whereas it is hard to precisely define potential cut-off in neurophysiological or clinical data in CTS, patients with mild symptoms admitted to surgery received preoperative conservative treatments, based on steroidal and non-steroid anti-inflammatory drugs, vitamin B6, local steroid injections, or hand braces. In patients with moderate and severe symptoms, surgical treatment is generally required. The first category comprises patients with slow nerve conduction but good muscle strength and pain usually worse during the night rather than the day. In case of failure of conservative treatments, surgery was offered.

Preoperative assessment: neurophysiology and ultrasonography

Clinical assessment is considered the gold standard, and in the presence of negative motor or sensory signs, collecting an accurate clinical history is mandatory for diagnosis. However, the real need for confirmatory testing and the role of nerve conduction studies, electromyography, and nerve ultrasonography in treatment decision-making is still debated. The electrophysiological assessment, particularly nerve conduction studies (NCS), is very sensitive to identifying a median nerve dysfunction, the degree of focal demyelination and/or axonal loss, and therefore it can quantify the nerve damage and its prognostic consequences [11].

**Figure 1:**
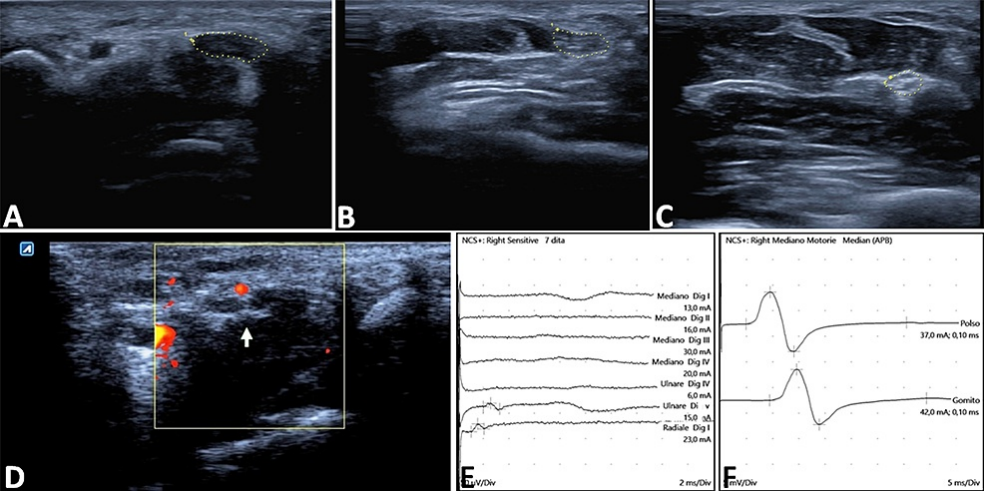
Preoperative assessment of median nerve entrapment The ultrasonographic scan of the median nerve shows a cross-sectional area (CSA) of 18 mm^2^ at the level of the pisiform bone (A), while more proximally, the median nerve (B) presents the classical “heart-shape” CSA. At the pronator quadratus level (C), the CSA and the echogenicity appear normal (CSA 8 mm^2^) with a wrist-to-forearm ratio >1.4 mm^2^. Ultrasonographic imaging could also identify the vascular structures by using the Doppler effect (D). The corresponding nerve conduction studies show a not recordable sensory conduction (E), with an increased distal latency of 4.9 ms (F).

Recently, the availability of probes that reach high frequencies allows reaching the resolution necessary for correctly visualizing superficial peripheral nerves and fascia. Ultrasonography (US) is a valid method for identifying an entrapment and a valid diagnostic aid for focal mononeuropathies. In CTS, the hallmarks of median nerve entrapment are present of a focal reduction of nerve size (cross-sectional area) at the site of compression and an increased nerve section proximal to the compression (Figure 1) [12,13]. These features sometimes are detectable before evidence of abnormality at the nerve conduction studies. In our experience, especially in more complicated cases where the diagnosis of CTS may require confirmation (i.e., in pre-existing cervical radiculopathy or sensory polyneuropathy or chronic tenosynovitis, or recurrent disease), US combined with NCS gives several advantages: the cross-sectional area, flattening ratio, and palmar bowing of the median nerve, along with information on nerve vascularity with power Doppler can confirm uncertain diagnoses. Furthermore, the US can show the presence of a bifid median nerve, an anomaly in which also a concomitant persistent median artery can be detected.

Surgical technique

**Figure 2:**
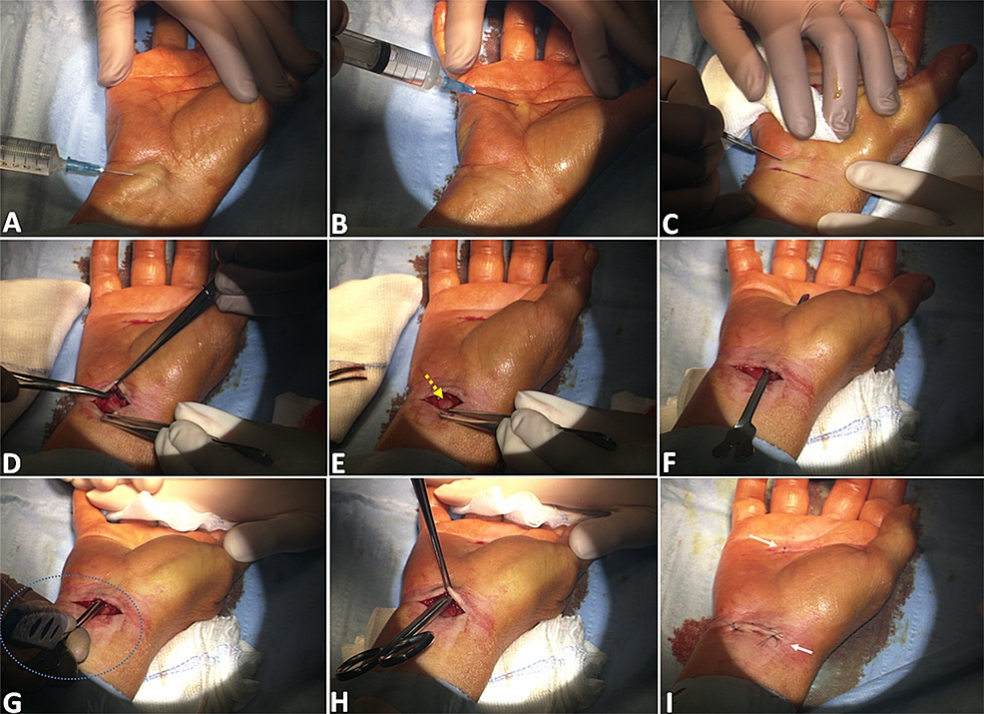
Step-by-step technique description After administering local anesthesia (5 ml of 0.5% lidocaine) both at the wrist (A) and in correspondence of the palmar incision (B), a small transverse incision (1 cm proximal to the wrist crease) is performed (C). Subcutaneous tissues are then dissected in a blunt way (D) to expose the median nerve at the entrance of the carpal tunnel and are visualized (yellow arrow in E), together with the proximal edge of the TCL and with the palmaris longus tendon. The grooved guide is inserted into the tunnel (F) and pushed forward beneath the ligament in a slightly medial direction (to avoid any median nerve branch that could originate at this level) to about 4 cm from the base of the fourth finger, and exit through a 3-mm incision made over the tip of the guide. The carpalotome (dotted blue circle) is finally inserted into the guide and moved forward along the groove (G): the ligament cutting creates a characteristic “grating” sound, determining an adequate carpal tunnel release (H). The two small incisions are sutured with 5-0 prolene (I).

All procedures were performed under local anesthesia based on injection of 5 ml of 2% lidocaine (Figure 2). A single, small, linear transverse incision proximal to the wrist fold is performed. After unsharpened dissection of subcutaneous tissues and antebrachial fascia, the median nerve is exposed, possibly with the site of origin of its superficial palmar branch if it is present. The proximal edge of the TCL and the palmaris longus tendon are visualized too. After the section of the proximal edge of the TCL, a grooved guide is inserted in a slightly medial direction towards the fourth finger. This strategy prevents possible damages of nerve branches that could originate at this level. A small incision over the guide’s tip allows a wide corridor in the context of the ligament. The carpalotome is then inserted into the guide and is moved forward along its groove until it stops due to the presence of the notch in the guide tip. The final result is a wide release of the nerve. The carpalotome and its guide are then retracted, and the two minor wounds are closed with 5-0 prolene sutures. The entire procedure requires less than 10 minutes.

## Results

From January 2001 to December 2020, 1568 operations on 1371 patients were performed at our institution using the described technique. The patients’ cohort showed a higher prevalence of women (68%), with a mean age of 56.4 years (range 24-88 years). Paresthesia and numbness of the first three fingers were the most frequent signs and symptoms, with a mean lasting of a minimum of three months. A smaller proportion of patients (23, representing 3.8% of the series) also showed various hyposthenia and muscle hypotrophy.

All patients were submitted to a preoperative electrophysiological evaluation, which revealed the typical signs of CTS in almost the entire cohort. The US evaluation of the median nerve at the wrist was a more recent introduction, dating from 2016; thus it has been employed in 289 patients only. In 37 patients, despite an electromyography (EMG) not showing marked neurophysiological signs of severe CTS, the ultrasonographic evaluation was strongly consistent with the clinical diagnosis. However, the clinical examination and the patients’ history determined, in such cases, a surgical indication. The carpal tunnel release determined, in this specific subcohort, the resolution of symptoms.

In 99.8% of total patients, we obtained a complete symptoms remission, with the disappearance of acroparesthesia and numbness since the early post-operative time. Patients with hypotrophy and hyposthenia showed few improvements in motor signs, as expected considering the prolonged degree of nerve damage leading to motor impairment. These results are comparable to the trend evidenced when we presented our institutional historical cohort in 2008 [7], comparing the double incision technique with the single wrist incision, always by using the carpalotome, with or without the assistance of the transillumination. Adding the second incision in the palm significantly increased the transverse ligament release. The mean duration of every single procedure was about 8 minutes. We experienced only four surgical complications, represented by wound infection not requiring a surgical correction but only systemic antibiotic treatment. Nevertheless, in 21 cases, patients experienced, after a transient amelioration, pain and paresthesias recurrence within three months; therefore, a second surgery was proposed to increase the TCL section.

## Discussion

Our technique demonstrated safety and effectiveness for median nerve release in CTS, with a low rate of adverse events and complications, reduced surgical time, and rapid return to hand movements. Despite the surgical treatments’ feasibility, CTS is a relevant condition regardless of the technique employed, considering its frequency and the long symptoms lasting before treatment. As a matter of fact, due to the chronic pain and the limitations to daily activities, CTS has a profound impact on brain structures, and the pain modulates the thalamus and insula networks, as recently demonstrated by Li et al. [14].

The epidemiology of CTS, its frequency mainly among the working population, and the different physicians treating such conditions (i.e., neurosurgeons, plastic surgeons, orthopedics, hand surgeons) led, during the years, to the flourishing of different techniques, aiming to reduce invasiveness and side effects as hypertrophic scarring, pillar pain, and prolonged convalescence. In the attempt to overcome the traditional “longitudinal technique”, one of the most promising strategies was the introduction of endoscopically assisted surgery, firstly described by Okutsu et al. in 1989 [15]. The endoscopic approach allows to examine the transverse carpal ligament and its adjacent structures directly. Several endoscopic-assisted techniques have been employed, whereas the most common is Chow’s dual-portal procedure [16]. Compared to open techniques, endoscopy is associated with the above-mentioned better results in a slightly higher percentage of cases [17]. However, the endoscopic release is a promising but challenging technique, presenting some disadvantages. The trocar placement is a blinded procedure; inserting a relatively large device through a narrow tunnel could lead to tissue damage before directly visualizing the anatomical structures. Moreover, often a tourniquet is used, which might lead to nerve ischemia if maintained too long. Obviously, an adequate learning curve is necessary, particularly for physicians who usually do not perform other endoscopic approaches than carpal tunnel release. On the contrary, open surgical techniques with small incisions as the mini-palmar longitudinal one [18], which is more anterior than the transverse one used in our technique, could determine an uncertain degree of tunnel release.

In our experience, using a carpalotome with two incisions can overcome some limitations of both endoscopic and mini-palmar techniques. This approach is based mainly on topographical anatomical landmarks. In fact, despite permanent injuries to the palmar cutaneous branch, thenar branch, and common digital nerves have been reported only in less than 0.12% of cases, the risk of nerve injury is higher in patients undergoing endoscopic CTR compared with open surgery [19]. However, in most cases, these damages are mainly temporary neurapraxias. On the contrary, in the attempt to avoid the branches of the median nerve, it must be avoided to direct instruments towards the ulnar side of the tunnel, increasing the risk of injury to the Guyon canal’s structures. Therefore, the direction we use to advance the grooved guide appears safe, also comparing the current results to our historical cohort (based on transillumination or mono-incision technique). In the previous series, damage of the primitive median artery requiring to switch to open technique was recorded [7], while no such side effect was due to the current approach. Whereas by using the abovementioned techniques a complete remission of acroparesthesia and numbness was obtained in about 90% of patients, 9.3% of patients in the historical cohort presented symptoms’ recurrence within two months, requiring a second surgery.

Finally, due to reduced invasiveness, we never used a tourniquet to control hemostasis. Considering our results, we think that the technique we adopted is an effective treatment for CTS: it combines the advantages of a low-cost, minimally invasive surgical technique with a reduced incidence of therapeutic failure.

Complications and other possible predicting factors

The groover guide and the carpalotome must be deep enough to avoid skin damage if the instruments are too superficial. Careful attention must be reserved to avoid the palmar cutaneous branch of the median nerve. The common digital nerves may be injured if surgical dissection is taken too far distally. Nerve damage could determine neuropraxia, persistent paresthesias, or painful neuroma. We also suggest particular attention to skin disinfection after surgery; we control the wound at first and tenth days after surgery in a hospital outpatient setting. We suggest patients to early move the hand, avoiding prolonged immobilization: no late hematomas have been encountered following hand movements.

In patients with NCS not conclusive but with an evident clinical syndrome, the use of US can confirm ultrastructural alterations that could indicate the need for surgical treatment. An ongoing trial is comparing, at our institution, the effectiveness and predictive value of preoperative US also in case of negative neurophysiological studies. Finally, in case of multiple recurrences or associated conditions such as lipomatosis of the nerve and non-syndromic macrodactyly, advanced imaging techniques such as MRI or diffusion tensor imaging (DTI) should be considered [20].

Limitations

The main limitation of our analysis is the retrospective nature, which could per se determine some bias compared to a prospective evaluation. Moreover, although preoperative electromyography and NCS were performed in all patients, their invasiveness and costs greatly limited a long-term post-operative evaluation, especially in patients with a satisfactory subjective outcome. In our opinion, the application of routine ultrasonographic evaluation could overcome these limitations.

## Conclusions

The carpalotome with double incision technique confirms representing a valid therapeutic strategy in CTR because it combines the advantages of a low-cost, minimally invasive surgical technique with a minimal incidence of therapeutic failure. A complete preoperative assessment based on neurophysiological and ultrasonographic evaluation provides reliable anatomical and functional information in the decision-making process, helping to prevent possible damages due to unexpected anatomical findings, thus limiting the recurrence of disease due to incomplete carpal tunnel section.
